# Supplementary material for: Beauveria bassiana (Hypocreales: Clavicipitaceae) Volatile Organic Compounds (VOCs) Repel Rhynchophorus ferrugineus (Coleoptera: Dryophthoridae)
Source: J Fungi (Basel). 2022 Aug 11;8(8):843. doi: 10.3390/jof8080843 (PMC9410273; doi:10.3390/jof8080843)
Supplement: Supplementary file 1 [file jof-08-00843-s001.zip › jof-1861381-supplementary.pdf]

Journal of
Fungi

MDPI

Supplementary Materials

Beauveria bassiana (Hypocreales: Clavicipitaceae) Volatile Organic Compounds (VOCs) Repel *Rhynchophorus ferrugineus* (Coleoptera: Dryophthoridae)

Johari Jalinas [1,2,3], Federico Lopez-Moya [1,*], Frutos C. Marhuenda-Egea [2,4] and Luis Vicente Lopez-Llorca [1,2]

1 Laboratory of Plant Pathology, Department of Marine Sciences and Applied Biology, University of Alicante, 03080 Alicante, Spain; johari_j@ukm.edu.my (J.J.); lv.lopez@ua.es (L.V.L.-L.)
2 Multidisciplinary Institute for Environmental Studies (MIES) Ramon Margalef, University of Alicante, 03080 Alicante, Spain; frutos@gcloud.ua.es
3 Department of Biological Sciences and Biotechnology, Faculty of Science and Technology, Universiti Kebangsaan Malaysia (UKM), Selangor 43600, Malaysia
4 Department of Agrochemistry and Biochemistry, University of Alicante, 03080 Alicante, Spain
* Correspondence: federico.lopez@ua.es

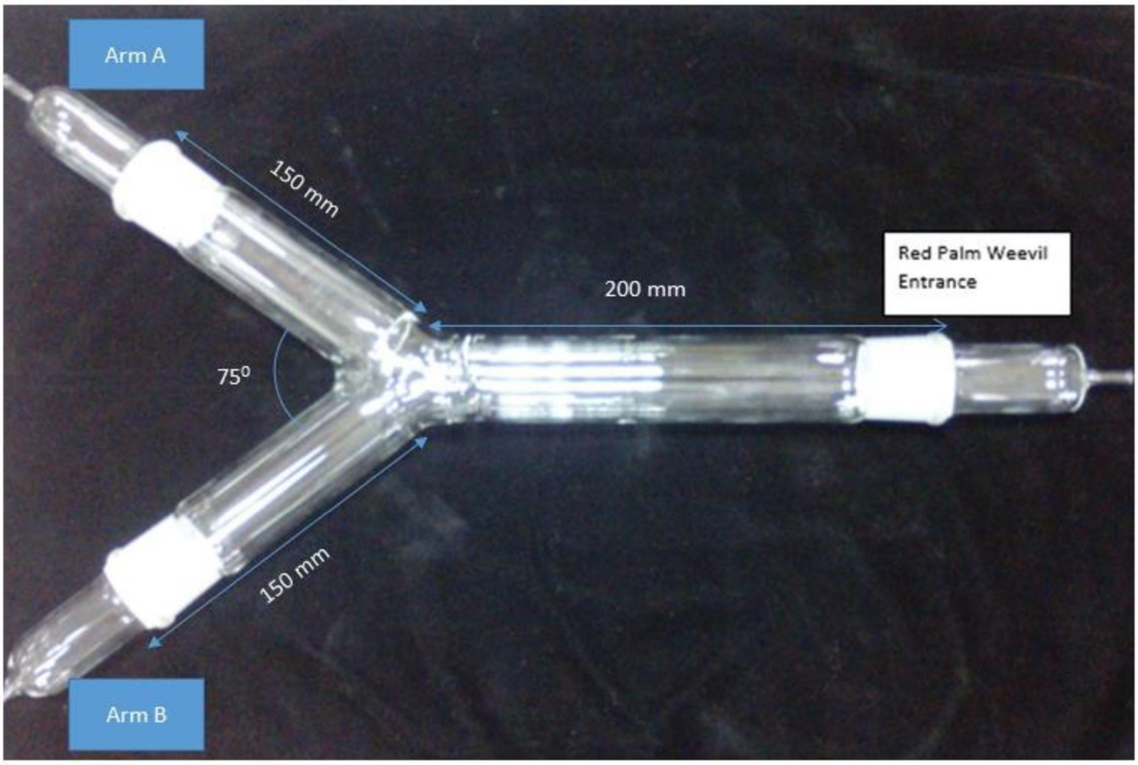

Figure S1. Tube Y olfactometer to test the response of adult red palm weevil (female) on different chemical stimuli.

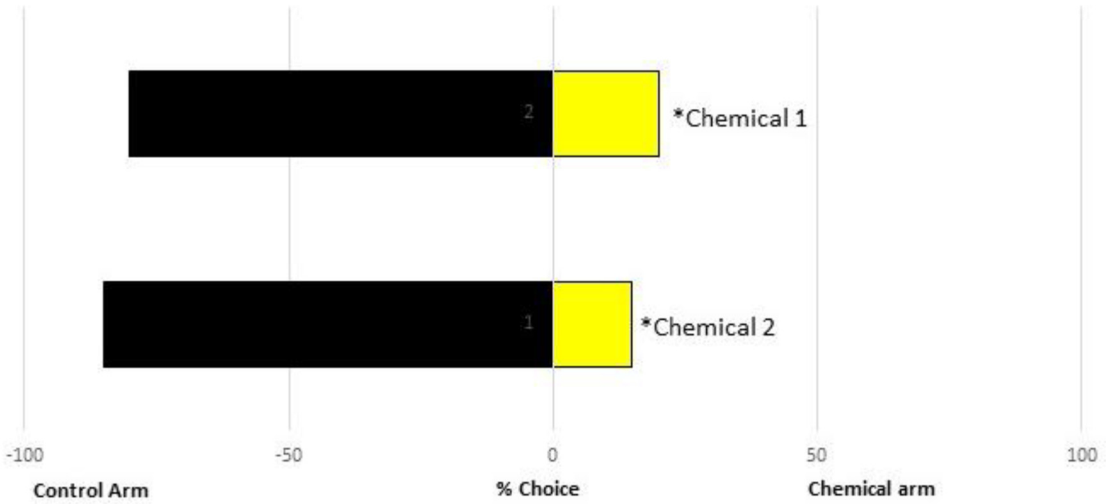

Figure S2. Response of a *Rhynchophorus ferrugineus* female in a Y-tube olfactometer (10 min) when given a choice between environmental air (control) and odour stimulus (Chemical stimuli) individuals per choice test. * Indicates significant difference within a choice test ($P < 0.01$, chi-square).
